# Supplementary material for: Use of unmanned aerial vehicles (UAVs) for mark-resight nesting population estimation of adult female green sea turtles at Raine Island
Source: PLoS One. 2020 Jun 4;15(6):e0228524. doi: 10.1371/journal.pone.0228524 (PMC7272060; doi:10.1371/journal.pone.0228524)
Supplement: S1 Table — (DOCX) [file pone.0228524.s002.docx]

| **Painted turtles** | **Survey period** | **Number surveys** | | |
| --- | --- | --- | --- | --- |
|  |  | **Surface observer** | **UWV** | **AUV** |
| 2000 | Dec 2013 | 6 | 1 |  |
| 1930 | Dec 2014 | 3 | 3 |  |
| 482 | Feb 2016 | 5 | 6 |  |
| 781 | Nov 2016 | 6 | 6 |  |
| 2000 | Dec 2016 | 6 | 5 | 3 |
| 2000 | Dec 2017 | 2 | 3 | 3 |
